# Sensitivity-informed framework for enrichment distribution in MNR for thermal performance enhancement

**DOI:** 10.1038/s41598-026-43564-y

**Published:** 2026-03-11

**Authors:** Umair Aziz, Hamda Khan, Zahid Hussain, Khalil Ullah, Imran Shah, Dong-Won Jung

**Affiliations:** 1 Department of Mechatronics Engineering, Air University, Islamabad, Pakistan; 2 Department of Sciences and Humanities, National University of Computer and Emerging Sciences, Islamabad, Pakistan; 3 Faculty of Applied Energy System, Major of Mechanical Engineering, Jeju National University, 102 Jejudaehak-ro, Jeju-Si, 63243 Republic of Korea

**Keywords:** Micro nuclear reactor, Radial power peaking, Fuel-clad mechanical interaction, FCMI, OpenMC, Non-uniform fuel distribution, Energy science and technology, Engineering

## Abstract

As interest in space exploration and remote power systems grows, Micro Nuclear Reactors (MNRs) are being explored for their compactness, long operational life, and inherent safety. However, challenges such as Radial Power Peaking (RPP), structural integrity, and fuel utilization under high-temperature conditions remain inadequately addressed. In this study, the issues of RPP, uneven power distribution, and related safety concerns have been analyzed. RPP occurs because neutron flux and subsequent fission rates decrease while moving from the core center toward the periphery. In the annular-fueled MNR core presented, a maximum power peaking factor of 1.28 is observed. RPP poses a significant design challenge as it induces geometric distortion, reduces the fuel clad gap, and may lead to Fuel Clad Mechanical Interaction (FCMI), which is treated as a design-basis operational constraint for long-term autonomous operation of the reactor presented in this study. Consequently, the designed 1 MWth core power must be reduced in proportion to the peaking factor, limiting the allowable operating power to 738 kWth to ensure local thermal-mechanical limits are not exceeded. This study proposes a novel, zone-based, sensitivity-informed non-uniform fuel enrichment framework that mitigates RPP without any physical design changes. For the annular-fueled MNR core, the strategy reduces RPP by 75%, enabling a 28.7% increase in safe operating power from 738 kWth to approximately 950 kWth.

## Introduction

Nuclear reactors with power output less than 20 MWe are classified as Micro Nuclear Reactors (MNR)^1,2^. This category of reactors includes diverse technological concepts including Light Water Reactors (LWR) and fast spectrum designs intended for high temperature, compact, and specialized applications. The reactor discussed in this study is a fast spectrum gas cooled reactor designed for specialized applications such as space exploration missions. These are compact reactors and can be completely manufactured at a factory and transported to the installation site^3,4^. They are Gen-IV reactors and are being designed with inherent passive safety features that will allow self-regulation^5^ and prevent meltdown^6,7^. The features have renewed the interest in nuclear energy^8^. Their compact nature also makes them suitable for integration with renewable energy sources. MNRs are modular energy systems that are suitable for off-grid installations, emergency energy restoration, and space exploration missions^9,10^. A key design feature is that they can operate for years with little to no human effort. However, MNRs have many design challenges, one of which is Radial Power Peaking (RPP). RPP occurs due to uneven power distribution in the core^11^. It not only limits the performance of the reactor but also poses a risk to structural integrity.

Conventional reactors address this issue through periodic refueling and fuel zoning strategies^12^. Fuel zoning is explored in this work as a systematic, sensitivity informed approach to mitigate radial power peaking. MNRs are being designed to operate for years without human intervention in space exploration missions and remote areas. In the reactor core, the peaking occurs in radial as well as in axial direction. This is because neutron flux and subsequent fission rates decrease while moving from the center towards the periphery^13^. RPP poses a greater risk as it can lead to a reduction in the fuel-clad gap due to fuel swelling and thermal expansion. In some cases, the geometric distortion of the fuel results in Fuel Clad Mechanical Interaction (FCMI). In conventional commercial reactors FCMI is a managed operational state. Avoiding contact is a design-based operational constraint for the proposed MNR due to its 10 years of autonomous operation in remote or space environments without human intervention. This prevents localized stress concentrations and progressive cladding strains, thereby ensuring long term structural reliability as Mo-Re cladding remains within the elastic regime throughout its mission life. Power peaking also affects the other design and operational parameters, such as cooling requirements, thus causing hotspots in the core. Several MNR core designs have been proposed over the years, and a few of those have discussed the issue of radial power peaking and mitigation strategies.

A lithium heat pipe-based MW thermal power reactor design has been presented by^5^. The use of passive cooling technology reduces the moving parts in the overall system. The system uses control drums to manage reactivity and maintain criticality. Such designs, however, are based on uniform fuel enrichment in the core, which leads to uneven power distribution in the core, localized hot spots, and radial power peaking^14^. Radial power peaking requires the use of mitigation strategies, including the use of variable cooling requirements or non-uniform fuel enrichment placement.

Westinghouse has also presented a heat pipe-based modular reactor design called Evinci^15^. It is designed for off-grid and commercial applications. The focus is to keep the design simple within a monolith. The monolith contains housing for fuel, heat pipes, and moderator. The design undergoes radial power peaking and requires mitigation strategies. A coupled neutronic and thermal-hydraulic study has been conducted by^16^. The study considered the operation of the reactor under various heat pipe failure scenarios. Performance of core materials under long-term thermal load, however, requires further research.

Performance of UN fuel under high power densities was carried out by^17^. The study carried out a transient analysis of thermal stress. Comprehensive power peaking analysis in fast reactor cores to enhance operational efficiency and reduce localized stress has received limited attention. Liu et al.^18^ conducted thermal-hydraulic design of a 500kWth MNR with 45% enriched UN fuel. The concepts of heat pipe thermal resistance and thermoelectric conversion have been explored. However, the role of enrichment zoning to mitigate uneven power profiles and improve thermal performance across the reactor core remains an area of ongoing interest.

Aziz et al.^19^ have presented the issue of RPP in a conceptual heat pipe cooled MNR core with solid fuel and have explored the use of non-uniform enrichment distribution as a viable power peaking mitigation strategy. However, a comprehensive strategy to effectively distribute enrichment in the core was not in the scope of the study. Fakhrul Islam^2^ has presented a conceptual design and preliminary safety analysis of a proposed nuclear microreactor for mobile applications. The gradual increase in fuel enrichment in the radial direction is explored and proposed as a viable strategy for mitigating the power peaking. However, a systematic approach that provides enrichment values for different regions of the core has not been extensively examined. Yuhao He et al.^20^ presented a comprehensive review of power flattening methods and neutron characteristics of a megawatt-class space gas-cooled fast reactor. The use of absorber material in fuel rods that exhibit power peaking was proposed, and a noticeable reduction in power peaking was observed.

It is found that the studies thus far have been largely carried out while considering the conventional periodic in-core fuel management for large thermal reactors, and the uniform distribution of the fuel for MNRs. Detailed investigation into power peaking and the effect of fuel location has received limited attention as a mitigation strategy in MNRs.

The research gaps identified are thus the lack of a comprehensive strategy to mitigate radial power peaking and limited thermo-mechanical analyses of the fuel clad interaction due to radial power peaking. These gaps form the foundation for this study, which addresses these challenges by proposing a novel framework. The proposed framework performs sensitivity analysis on the reactor core; the sensitivity analysis data is used to assign weightages to different regions of the core for the fuel’s sensitivity to reactivity. A one-time non-uniform fuel enrichment distribution strategy is then suggested by the framework that mitigates the power peaking and enhances the operating power of the core. The novelty of this framework lies in the ability to mitigate RPP through effective fuel placement and sensitivity-informed enrichment distribution, thereby eliminating the need for any physical modification to the core geometry.

## Materials and methods

### Reactor core

**Fig. 1:**
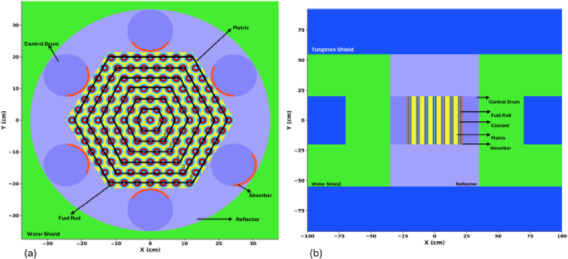
MNR Core (**a**) top view (**b**) side view.

**Fig. 2:**
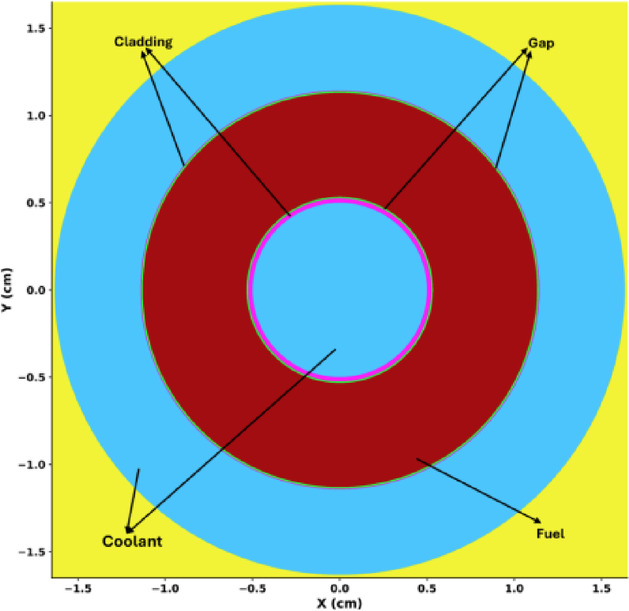
Annular fuel with coolant flowing through annulus and outer channel.

The reactor core is a fast spectrum Highly Enriched Uranium (HEU) core with 70% enriched Uranium Nitride (UN) fuel. The enrichment level is significantly higher than the civil Low Enriched Uranium (LEU) or High Assay Low Enriched Uranium (HALEU) ranges; however, it is consistent with the KRUSTY^21^ experiment and was selected to achieve the compactness and high power density required for the long duration autonomous operations, such as space exploration, where refueling is not feasible. Accordingly, the core is designed for a cycle length of 10 years of continuous and autonomous operation. OpenMC^22^ has been used to model the conceptual hexagonal 1 MW thermal MNR core with housing for fuel rods. The top view and side view of the modeled core, along with its shields and control drums for reactivity control, are shown in Fig. 1 (a) and Fig. 1 (b), respectively. The core is surrounded by the reflector, control drums, and radial and axial shields for neutrons and gamma rays. Because the system is a fast spectrum reactor, it does not use a moderator for its core operation. However, a layered shielding strategy is incorporated to ensure protection of structural components and surrounding systems. Tungsten shield is utilized for gamma attenuation and fast neutron scattering, while the water shield is used for neutron moderation and absorption. The shielding layers are shown in Fig. 1 (b). The reactor core contains 127 annular fuel rods, shown in Fig. 2.

**Table 1:** Core materials.

Core Element	Material
Fuel	Uranium Nitride (UN)
Coolant	Helium (He)
Cladding	Molybdenum-Rhenium (Mo-Re)
Matrix	Niobium-Zirconium (Nb-1Zr)
Reflector	Beryllium oxide (BeO)
Absorber	Boron Carbide (B4C)
Shields	Tungsten, Water

Heat generated by nuclear fission is removed by coolant flowing through the central annulus of each rod and the surrounding external coolant channel. The extracted heat can be used by TEG to generate electricity directly from heat. There are six control drums made of the reflector material BeO. These control drums are lined with absorber boron carbide (B4C) on one side. The movement of control drums and absorbers with reference to the core (absorber facing towards or away from the core) is used to maintain criticality and manage excess reactivity.

The motivation for using annular fuel in the design is its dual-channel heat removal capability, which enhances thermal efficiency and safety. Studies^23–25^ have shown that annular fuel can achieve a 30–50% increase in heat removal efficiency compared to solid fuel, depending on the reactor design and operating conditions. This improvement not only enhances thermal performance but also reduces the risk of fuel damage under high-power or transient conditions. The gap between the fuel and cladding is kept at 100$\mu m$, allowing for a total expansion margin of 200$\mu m$ i.e., 100$\mu m$toward the inside and 100$\mu m$ toward the outside. Table 1 lists the key materials used to model the core, with their thermophysical properties obtained from^19^.

### Core criticality

To establish the feasibility of the design, the first step is to ensure that the core is critical and can sustain the chain reaction. To check the criticality of the core, Monte Carlo (MC) simulations were carried out using the OpenMC code^22^. The MC simulations were carried out for ${10}^{5}$ histories and 10,000 batches on a system with Intel(R) Core (TM) i7-11800 H @ 2.30 GHz and 64 GB RAM. All simulations in this work have been carried out using OpenMC v0.15.1 with ENDF/B-VIII.0 cross-section libraries.

The effective multiplication factor, ${k}_{eff},$ for 70% enriched fuel with all but one control drum facing the core at the beginning of life is estimated as 1.00085 with a relative error of $\pm0.000081.$ The ${k}_{eff}$, when all absorbers are facing towards the core and are facing away from the core, is estimated as 0.95563 $\pm$ 0.00006 and 1.11856 $\pm$ 0.00004, respectively. The reactor is expected to operate for 10 years without refueling. The beginning of life (BOL) excess reactivity $({k}_{eff}\cong1.118)$ provides a sufficient reactivity bank of ~10,550 pcm which compensates for the fuel burnup over reactor life. This margin is consistent with the values reported in literature where margins in the range of 5000–12,000 pcm are shown to sustain similar multi-year operation^26,27^. Due to the fast spectrum, the reactivity swing is expected to be minimal, ensuring the core remains critical throughout the intended mission. Thus, the reactor criticality, shutdown margin, and excess reactivity are established for the proposed design.

### Heat removal

The coolant used to remove the heat generated by the fuel rod can be a gas or a liquid metal. The principle of conservation of energy, given as Eq. 1, states that:

1 $$\dot{Q}_{R} = \dot{m}_{R} C_{p} \left( {T_{{out}} - T_{{in}} } \right) = \dot{m}_{R} C_{p} \Delta T$$

where ${C}_{p}$ is the specific heat of the coolant, ${\dot{m}}_{R}$ is mass flow rate, ${\dot{Q}}_{R}$ is thermal power, and ${T}_{out}$, ${T}_{in}$ are the outlet and inlet temperatures of the coolant, respectively.

The majority of thermal energy produced in nuclear fuel is derived from the kinetic energy of fission fragments. Consequently, the amount of heat generated per unit volume is proportional to the fraction of fissionable nuclear fuel consumed over a specific period. The linear power generation in a fuel rod typically varies with its position within the reactor, due to changes in the neutron flux distribution.

Heat removal from the inner and outer cladding surfaces was modelled using the effective convective heat transfer boundary conditions. A convective heat transfer coefficient of $h=400\frac{W}{{m}^{2}K}$ was applied which represents a conservative, low to moderate forced helium convection in gas cooled reactor channels for the conceptual micronuclear reactor, where the thermally induced stress and deformations are of primary interest^28,29^. Since the analysis focuses on the thermo-mechanical response, pressure fields including system pressure and rod fill pressure were not included as mechanical loads.

### Solid mechanics

HEU MNRs operate at high temperatures, which induce thermal stresses in the fuel, cladding, and other core components. Over long periods, these stresses may cause deformation in the fuel. The hoop$\left({\sigma}_{\theta}\right)$, radial$\left({\sigma}_{r}\right)$, and axial stresses $\left({\sigma}_{z}\right)$ in a long hollow cylinder with an inner radius ${R}_{1}$ and an outer radius ${R}_{2}$ can be expressed by Eqs. 2, 3, and 4. These expressions reduce to the solid-cylinder form when ${R}_{1}\to0$^30^:

2 $${\sigma}_{r}=\frac{E\alpha}{1-\nu}\left[\frac{{R}_{1}^{2}}{{r}^{2}}\frac{{r}^{2}-{R}_{2}^{2}}{{R}_{2}^{2}-{R}_{1}^{1}}{\int}_{{R}_{1}}^{{R}_{2}}Trdr-\frac{1}{{r}^{2}}{\int}_{{R}_{1}}^{r}Trdr\right]$$

3 $${\sigma}_{\theta}=\frac{E\alpha}{1-\nu}\left[\frac{1}{{r}^{2}}\frac{{r}^{2}-{R}_{1}^{2}}{{R}_{2}^{2}-{R}_{1}^{1}}{\int}_{{R}_{1}}^{{R}_{2}}Trdr+\frac{1}{{r}^{2}}{\int}_{{R}_{1}}^{r}Trdr-T\right]$$

4 $${\sigma}_{z}=\frac{E\alpha}{1-\nu}\left[\frac{2}{{R}_{2}^{2}-{R}_{1}^{2}}{\int}_{{R}_{1}}^{{R}_{2}}Trdr-T\right]$$

The total strain can be calculated by using generalized Hooke’s law given in Eqs. 5, 6, and 7^31^.

5 $${\epsilon}_{r}=\frac{1}{E}\left[{\sigma}_{r}-\nu\left({\sigma}_{\theta}+{\sigma}_{z}\right)\right]+\alpha\left(T-{T}_{o}\right)+{\epsilon}_{r}^{s}+{\epsilon}_{r}^{c}$$

6 $${\epsilon}_{\theta}=\frac{1}{E}\left[{\sigma}_{\theta}-\nu\left({\sigma}_{r}+{\sigma}_{z}\right)\right]+\alpha\left(T-{T}_{o}\right)+{\epsilon}_{\theta}^{s}+{\epsilon}_{\theta}^{c}$$

7 $${\epsilon}_{z}=\frac{1}{E}\left[{\sigma}_{z}-\nu\left({\sigma}_{r}+{\sigma}_{\theta}\right)\right]+\alpha\left(T-{T}_{o}\right)+{\epsilon}_{z}^{s}+{\epsilon}_{z}^{c}$$

where $E$ is Young’s modulus, $\alpha$ represents the coefficient of thermal expansion, $\nu$ is Poisson’s ratio, $T$ represents the temperature at radial position $r,{T}_{0}$ represents the reference temperature, ${\epsilon}^{s}$ and ${\epsilon}^{c}$represent the swelling and creep strain, respectively. The thermal creep for UN fuel is given as Eq. 8^32^:

8 $$\dot{\epsilon}=2.054\cdot{10}^{-3}{\sigma}^{4.5}{e}^{-\frac{39369.5}{T}}\cdot\left(\frac{0.987{e}^{-8.65Por}}{{\left(1-P\right)}^{27.6}}\right).$$

The creep activation energy is $Q=327160\frac{J}{mol}=78.2\frac{kcal}{mol}.$

The thermo-mechanical analysis was performed in COMSOL^®^ Multiphysics, assuming quasistatic conditions and isotropic material properties. The COMSOL^®^ model is initialized at a reference temperature of 293 K representing the stress-free state. Thermal boundary conditions consist of bulk coolant temperature at 600 K and a convective heat transfer coefficient of 400 $\frac{W}{{m}^{2}K}$ for the convective heat removal from the cladding surfaces. These boundary conditions drive the radial temperature gradients and associated stress presented in Sect. “Fuel rod temperature and radial displacement”.

### Power distribution using OpenMC

MC simulations were carried out using OpenMC for multiple drum configurations, including all control drums facing towards, facing away from the core and mixed configuration that corresponds to the BOL, and fission rates were tallied. To study the power distribution in the core, the configuration with all control drums facing away from the core is adopted. Although this is not an operational state, this configuration provides a consnervative bounding condition that maximizes the local power density and thermal loading while preserving the hexagonal symmetry. It represents the most limiting condition for assessing radial power peaking. Due to the hexagonal nature of the core, there is a 1/6th symmetry in the core; therefore, the results have been presented for 1/6th of the core. However, all simulations were carried out for the full core.

### Non-uniform fuel enrichment distribution

In nuclear reactors, use of uniformly enriched fuel results in an uneven distribution of power as it does not account for the radial flux gradient. Uneven power distribution results in uneven fuel burnup, which reduces the overall reactor life. Fuel enrichment can, therefore, play a crucial role in balancing the neutron distribution in the core as well as maintaining criticality. In this study, a structured framework is developed that uses the relative sensitivity of different regions of the core to unevenly distribute the fissile isotope. This uneven distribution of fissile isotope is the non-uniform enrichment distribution. The non-uniform enrichment distribution compensates for the radial flux gradient and subsequent power distribution, as fission rate depends on both the neutron flux and spatially varying fissile isotope density. The framework relies on the relative sensitivity of different regions of the core to redistribute the fuel enrichment in a way that keeps the reactor critical but mitigates radial power peaking. The enrichment distribution is based on the following:

i. Sensitivity analysis: identification of core regions (hexagonal fuel rings) with the highest sensitivity to enrichment changes.
ii. Fuel rod density: varying number of fuel rods in hexagonal fuel rings.
iii. Fuel ring weightage: integration of fuel rod density and fuel ring sensitivity.

This approach results in an informed enrichment distribution for each ring in the MNR core. It relies on the sensitivity of a ring coupled with the volume of fuel in that ring to assign a weightage or importance to each ring. The result is a flattened power distribution in the core, which minimizes the hotspots and improves overall performance. These concepts are further explored in the sections below.

#### Sensitivity function

The MNR core discussed in this study consists of 6 hexagonal rings with a different number of fuel rods. Each of these plays a different role in maintaining the overall reactor criticality through its relative position from the center or periphery. A sensitivity function, given in Eq. 9, is introduced to quantify this impact.

9 $${S}_{i}=\frac{\partial{k}_{eff}}{\partial{E}_{i}}$$

where ${S}_{i}$ is the sensitivity coefficient for fuel ring *i*, ${k}_{eff}$ is the effective neutron multiplication factor, and ${E}_{i}$is the fuel enrichment in ring *i.*

The sensitivity function links the change in effective multiplication factor to the change in enrichment. For rings with the most sensitivity, even a small change in enrichment results in a sharp change in reactivity. Therefore, the sensitivity coefficient quantifies the potential for modifying the flux profile; regions with high sensitivity provide the greatest leverage for the redistribution strategy, while regions with low sensitivity allow for enrichment changes with minimal impact on criticality.

#### Impact of fuel rods

Another factor that impacts the reactivity and flux behaviour in the core is the volume of fuel in a particular region, or fuel rings in the case of HEU MNR. This corresponds directly to the number of fuel rods in each ring. To accommodate the number of fuel rods, a fuel rod influence factor is introduced and is given as Eq. 10:

10 $${R}_{i}=\frac{{N}_{i}}{{\sum}_{j=1}^{N}{N}_{j}}$$

where ${N}_{i}$is the number of fuel rods in ring *i*, ${\sum}_{j=1}^{N}{N}_{j}$ is the total number of fuel rods across all rings and ${R}_{i}$represents the normalized rod density contribution in ring *i.*

#### Sensitivity weight

Finally, a weightage is assigned to each fuel ring based on the sensitivity function and fuel rod influence factor. The weightage of a fuel ring is defined as Eq. 11.

11 $${W}_{i}=\frac{{S}_{i}{R}_{i}}{{\sum}_{j=1}^{N}{S}_{j}{R}_{j}}$$

where ${W}_{i}$ is the enrichment weightage factor for ring *i.*

A higher weight is assigned to more sensitive rings, whereas a lower weight is assigned to less sensitive fuel rings.

#### Enrichment distribution

The enrichment is then redistributed using Eq. 12:

12 $${u}_{i}=\frac{\left({W}_{i}\cdot{E}_{avg}\cdot{\sum}_{j=1}^{N}{N}_{j}\right)}{{N}_{i}}$$

where ${E}_{avg}$ is the mean enrichment level across all fuel rings. This is also the enrichment level for a critical core with uniform enrichment. Equation 12 ensures that the rod weighted core average enrichment remains unchanged by enforcing a normalization constraint on the enrichment redistribution parameter ${u}_{i}$, which represents the relative enrichment adjustment applied to each fuel rod based on local power sensitivity. This preserves the original total fissile inventory while flattening the radial power profile through spatial redistribution.

### Structured framework for enrichment distribution

A structured framework is then developed that incorporates the non-uniform enrichment distribution strategy discussed in Sect. “Non-uniform fuel enrichment distribution”. The framework provides a step-by-step methodology to mitigate radial power peaking in fast-spectrum micronuclear reactors. The framework is thus suitable for single-batch core geometries and reactor designs. The framework is formalized in six steps as discussed below:

***Step 1: Establish Baseline Criticality with Uniform Enrichment***.

First step is to model the core using OpenMC and establish that the core is critical at a uniform enrichment level. In this study, the MNR is a compact 70% enriched fast-spectrum reactor. A baseline flux and power distribution profile with a uniformly enriched core is established.

***Step 2: Thermo-Mechanical Performance Evaluation***.

COMSOL^®^ simulations are carried out to ensure that the core modeled in step 1 does not exceed the thermo-mechanical limits at the desired power levels. For UN fuel the safety limit is the decomposition temperature of 2000 K [33,34] whereas for the Mo-Re cladding a conservative yield strength limit of ~ 350 MPa is adopted based on the experimental data for Mo-Re alloys [35,36].

***Step 3: Radial Power Peaking Analysis***.

An analysis of neutron flux and fission power density is carried out to identify the localized hotspots. In MNR, core power peaking is observed at the center of the core as well as at the periphery. The peaking at the center occurs due to high neutron density. In the radial direction, the neutron density decreases due to more leakage to the surrounding regions. However, in fast spectrum MNR designs, the core is surrounded by a reflector, thus peaking is observed at the periphery due to reflected neutrons. The power peaking at the periphery is a characteristic feature of the fast spectrum cores surrounded by a reflector [37,38]. Neutron leakage from the core is reduced due to efficient fast neutron reflection and neutron multiplication via (n, 2n) reactions in BeO at the core-reflector interface. Due to this, the fast neutron flux is enhanced at the outer ring which results in increased fission rates near the periphery.

***Step 4: Sensitivity-Informed Peaking Mitigation via Enrichment Redistribution***.

***Step 4a: Monte Carlo Sensitivity Analysis***.

OpenMC reruns are carried out to identify the relative sensitivity of the fuel rings. A Python script has been developed to automatically vary the material properties for OpenMC reruns.

***Step 4b: Weight-Based Enrichment Redistribution Algorithm***.

Using the sensitivity data generated in step 4a, relative weights are assigned to fuel rings while accounting for the number of fuel rods in each ring through a MATLAB code. The enrichment redistribution function, another MATLAB program, then calculates the enrichment values for each ring.

***Step 5: Reverification of Criticality***.

MC simulation is carried out with a non-uniform enrichment distribution to verify core criticality. This step only requires changing the material properties, i.e., the enrichment value, and does not require any change in the geometry of the core. This is a key feature of this framework that allows mitigation of radial power peaking without a design change.

***Step 6: Reanalysis of Power Distribution and Thermal Limits***.

**Fig. 3:**
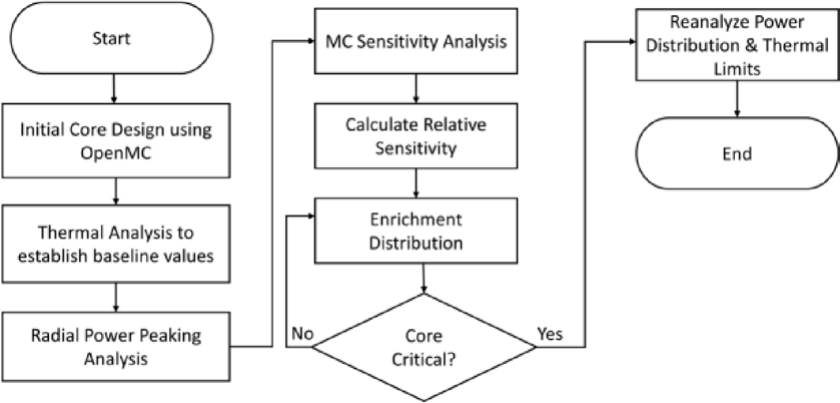
Framework for non-uniform fuel distribution.

In this step power distribution is re-analyzed to confirm and identify the mitigation of radial power peaking. Follow-up COMSOL^®^ simulations are also carried out to ensure that no new temperature peaks or stress points have emerged. This step ensures that the enrichment distribution strategy is neutronics wise and thermally viable. It is, however, important to note that a perfectly flat flux is not possible due to the fundamental nature of neutron production and leakage characteristics. The methodology outlined in this framework is presented in Fig. 3.

The framework outline in this section is not specific to a single MNR, but it can be applied to other single-batch configurations. The framework incorporates sensitivity of the core regions in a structured way to mitigate the power peaking, improve fuel utilization, improve reactor life, and enhance the reactor performance.

## Results and discussion

### Radial power peaking

**Fig. 4:**
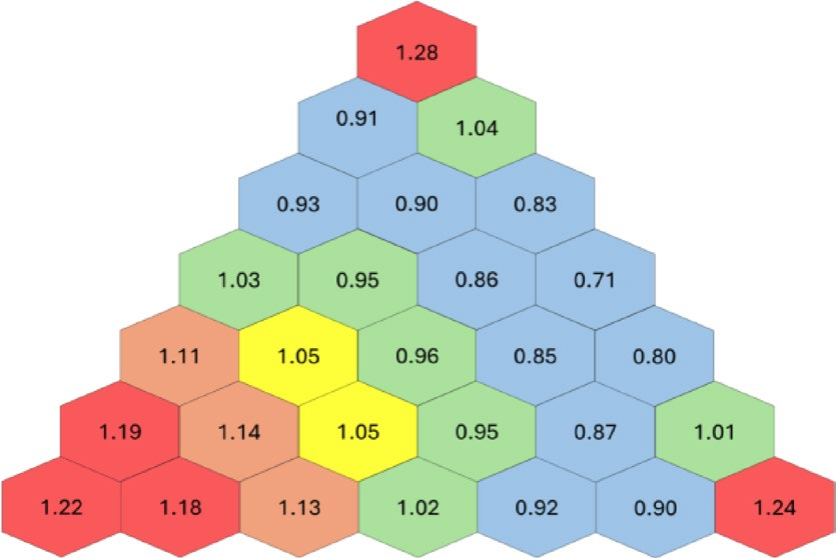
Power peaking factors.

For a 1 MW thermal reactor, the power requirement from each fuel rod is 7.87 kWth. An analysis of fission rates shows an uneven power distribution in the core. Radial Power Peaking Factors (PPF) for 1/6th of the hexagonal core are shown in Fig. 4. Significant peaking is observed at the center and periphery. A maximum power peaking factor of 1.28 is observed at the periphery. Peaking at the periphery is observed due to proximity to the reflector. It is also observed that several fuel rods operate at levels that are significantly lower than the designed power levels. This uneven distribution of power in the core leads to localized hotspots or power peaking. The fuel rod with the highest peaking factor operates at 10.08 kWth instead of 7.87 kWth due to localized neutron production and leakage characteristics. It is found that due to this peaking factor, the displacement in the fuel exceeds the fuel-clad gap of 100$\mu m$ on the outer surface (as shown in Fig. 7). Thus, to avoid Fuel-Clad Mechanical Interaction (FCMI) and any structural damage or fuel rupture, the 1 MWth core can only operate at 738 kWth.

### Fuel rod temperature and radial displacement

The fuel rod was analyzed to determine maximum temperature and geometric distortion using COMSOL^®^ Multiphysics. The model incorporated detailed geometry and material properties with explicitly defined boundary conditions, including a stress-free reference temperature of 293 K and steady state convective heat transfer boundary conditions applied to the cladding surfaces with a bulk coolant temperature of 600 K and a heat transfer coefficient of 400 $\frac{W}{{m}^{2}K}$. The power distribution obtained from the OpenMC was applied as a volumetric heat source within the fuel. Interaction between materials was modeled through conductive boundary conditions. The coupled solid mechanics and heat transfer physics, along with thermal expansion multiphysics, govern the resulting thermal strain and geometric distortion induced by the thermal load.

**Fig. 5:**
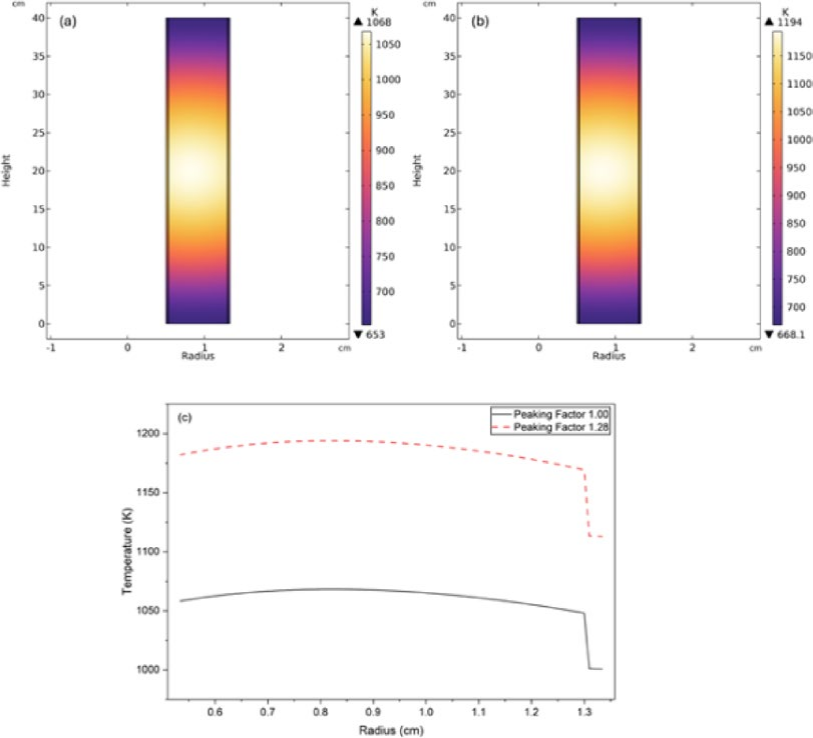
Fuel rod thermal profile (**a**) baseline power level (**b**) at maximum peaking factor (**c**) radial temperature profile at the axial center.

For a 1 MW thermal reactor, the baseline power for each fuel rod is 7.87 kWth, while the fuel rod undergoing maximum power peaking operates at 10.08 kWth. Figure 5. shows the temperature distribution in the fuel rod for baseline power level and the maximum power peaking condition. From Fig. 5a and b it can be seen that the maximum power temperature occurs at the axial center of the fuel. The fuel word at baseline power level reaches a peak temperature of 1068 K while the temperature in fuel rod at maximum peaking condition rises to 1194 K. In both cases the temperature remains well within the material limit of UN fuel which undergoes decomposition at 2000 K. This result confirms the thermal stability of the fuel at high-power densities. Figure 5c shows the radial temperature distribution in the fuel for both power levels at its axial center. The figure Fig. 5c also shows the sharp dip in the temperature across the fuel-clad gap due to low thermal conductivity of helium.

**Fig. 6:**
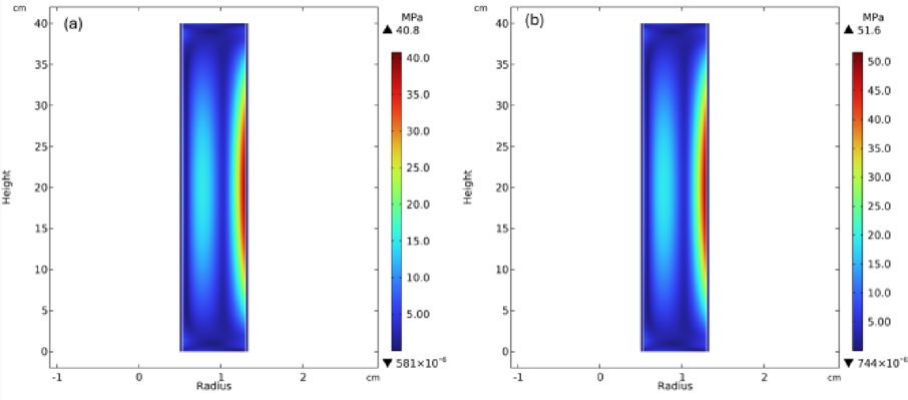
von Mises stress profile (**a**) baseline power level (**b**) at maximum peaking factor.

Figure 6a shows the von Mises stress distribution in the fuel rod for the baseline power level (7.87 kWth) and Fig. 6b shows the von Mises stress at maximum power peaking level (10.08 kWth). For baseline power, the maximum von Mises are 40.8 MPa while for the maximum power peaking the highest von Mises are 51.6 MPa on the outer surface of the fuel pellet. In contrast, the stress in the Mo-12Re cladding at the axial center is found to be negligible at 0.46 MPa. It is observed that for both power levels, von Mises remains well within the reported fracture limits of UN fuel and below the yield strength of Mo-Re cladding [32,36]. The fuel rod experiences maximum stress at the axial center which is also the region of highest thermal loading as shown in Fig. 5.

**Fig. 7:**
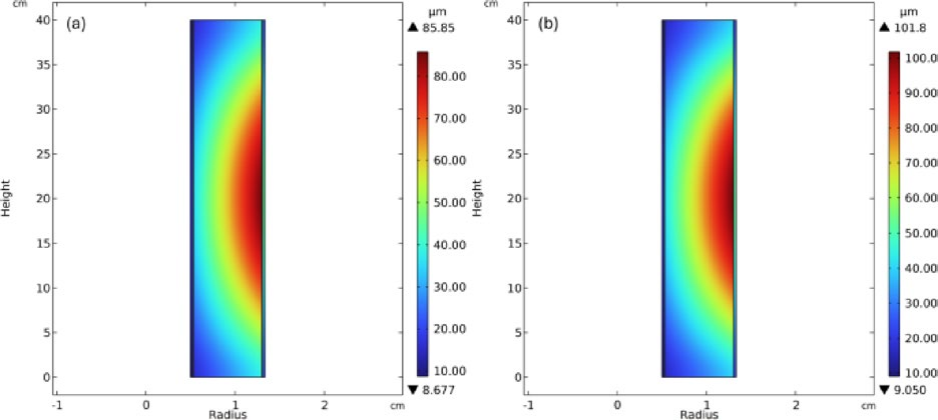
Radial displacement (**a**) baseline power level (**b**) at maximum peaking factor.

Figure 7. shows the radial displacement of the fuel rod for two power levels, i.e., 7.87 kWth and 10.08 kWth. Where 10.08 kWth is the power level of the fuel undergoing the highest power peaking of 1.28. When the fuel rod is operating at the designed 7.87 kWth power, the maximum displacement of 85.85$\mu m$ is observed at the axial center of the fuel pellet on the outer surface as shown in Fig. 7a. The displacement of 85.85$\mu m$is within the allowed limits when a fuel rod is operating at the desired power level. However, when a fuel rod is operating at a higher than desired power level due to radial power peaking, the pellet displacement exceeds the fuel-clad gap. Figure 7b shows the radial displacement for a fuel rod with a peaking factor of 1.28, which operates at 10.08 kWth. The maximum displacement at this power level is 101.8$\mu m$ at the outer surface, which exceeds the designed fuel-clad gap. This result shows that radial power peaking leads to fuel-clad mechanical interaction at the core’s thermal hotspots, which can potentially compromise the structural integrity of the core over long-term operation.

Although the von Mises stress remains low at fuel-clad interaction, exceeding the $100\mu$m fuel-clad gap is treated as a design-basis operational limit for the present micronuclear reactor concept, rather than a generic safety violation. This is to avoid prolonged FCMI due to the fast spectrum long term autonomous operation. Long term exposure to FCMI can lead to progressive creep, non-uniform swelling, altered gap conductance, and accelerated material degradation. Therefore, mitigation of radial power peaking is critical for several operational factors beyond immediate structural integrity. Flattening the power profile ensures uniform fuel burnup and extends the operational lifespan by minimizing localized fission gas release and swelling. Furthermore, it restores thermal-hydraulic margins that would otherwise be limited by the hottest fuel rods, directly enabling the 28.7% increase in total core power capacity of the proposed reactor. This improvement corresponds to the relative increase in the total power that the optimized core can sustain with the non-uniform enrichment distribution as compared to the baseline uniform core, before violating the design-based operational limit of fuel-clad interaction. This is further explained in Sect. “Power peaking mitigation”.

### Power peaking mitigation

An analysis of the power distribution from Fig. 4. shows that power peaking occurs at the center of the core and at the periphery. These regions correspond to rings 1, 2 at the center of the core and ring 6 at the periphery. However, rings 3, 4, and 5 are identified as the most sensitive rings. This is attributed to the localized neutron leakage to the surrounding regions, causing a significant drop in the local fission rate. Therefore, the central rings 3, 4, and 5 (not the actual center of the core) require more fissile isotope as compared to the inner rings (rings 1 and 2) and outer ring (ring 6) to compensate for flux depression. Due to low sensitivity, the enrichment value in rings 1, 2, and 6 can be reduced without affecting the reactor criticality. This reduction in enrichment also contributes to flattened power distribution.

**Fig. 8:**
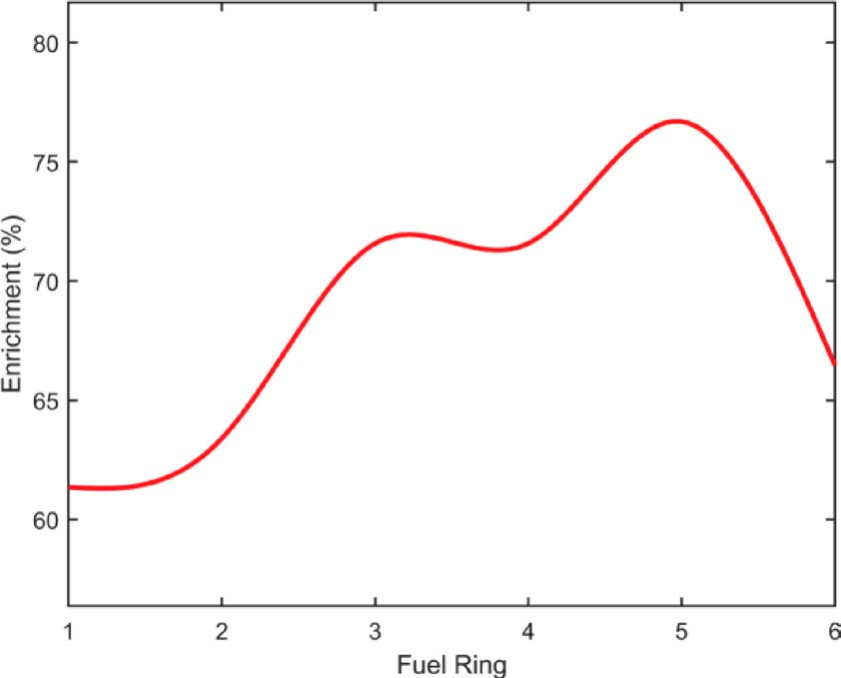
Non-uniform fuel enrichment distribution.

The nonuniform fuel enrichment distribution for the core was thus calculated using the framework presented in Sect. “Non-uniform fuel enrichment distribution”. The non-uniform enrichment from ring 1 to ring 6 is calculated as 61-63-71-71-76- 66%, respectively. Figure 8. shows the non-uniform fuel enrichment for each ring. To verify whether the core remained critical with this enrichment distribution, Monte Carlo simulations were performed using OpenMC. The results confirmed reactor criticality, with ${k}_{eff}=1.00008+/-0.00026$, validating the non-uniform fuel distribution strategy.

**Fig. 9:**
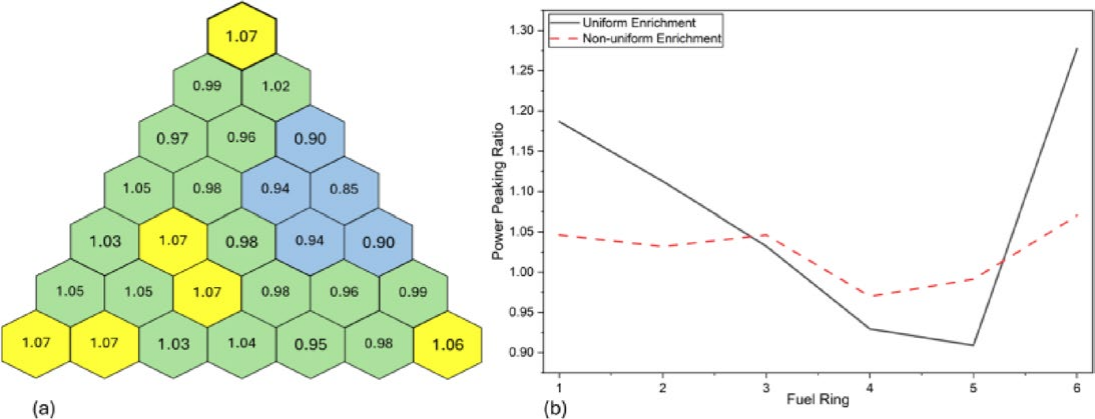
Power distribution with non-uniform enrichment distribution (**a**) for each fuel rod (**b**) per ring.

To further establish the advantages of non-uniform fuel distribution, the fission rates were re-analyzed. As shown in Fig. 9a and b, the hotspots observed in the core with uniform enrichment (Fig. 4) are significantly reduced. The maximum peaking factor was reduced from 1.28 to 1.07, resulting in a more even power distribution across the core without any physical design change in the core geometry. The core is now able to operate at 949.9kWth as compared to 738.6kWth with uniform fuel distribution, an enhancement of 28.7%.

## Conclusions

Micro Nuclear Reactors (MNR) are Gen-IV reactors that can be fabricated at a factory and transported to the installation site. Uneven power distribution in the core results in localized hotspots. This phenomenon occurs both radially and axially as the neutron flux and fission rate decrease while moving from the center of the core towards the periphery. The MNR core presented in this work is surrounded by a BeO reflector. Due to this reflector, the radial power peaking occurs at the center of the core as well as at the periphery. Several fuel rods in the core exceed the $100\mu m$ fuel-clad gap adopted as a design-basis operational limit for the proposed MNR. To ensure long-term reliability during autonomous operation, the prolonged fuel clad interaction is intentionally avoided, even though fuel clad contact is not a generic safety violation. Consequently, without power peaking mitigation, the core must operate at the power level reduced by a factor equal to the peaking factor of 1.28. This translates to an operating power of 738kWth instead of 1MWth. MNRs are low-power systems, and reducing the operating power by over 26% is a challenge.

This study presents a novel framework that provides a strategy for fuel enrichment distribution in the MNR core based on a zone’s sensitivity $\left( {\frac{{\delta k}}{{\delta \varepsilon }}} \right)$ or weightage. This non-uniform distribution of the fuel enrichment in the core mitigates the effects of radial power peaking in the core and helps in achieving a flat power distribution in the core. OpenMC, an open-source particle transport code, has been used for neutronics of the core. COMSOL^®^ Multiphysics has been used to establish temperature profiles and geometric distortion in the fuel due to high-temperature operation.

A comprehensive sensitivity analysis was carried out to understand the impact of different fuel regions/zones of the core on reactivity and ${k}_{eff}$. A Python script has been written to automate the OpenMC re-runs that generate the necessary data required for the sensitivity analysis. A MATLAB™ code then uses the data from OpenMC re-runs to assign weightages to the fuel zones of the core. Another MATLAB™ function then proposes a non-uniform fuel enrichment strategy that mitigates the effects of radial power peaking.

Initially, the core had a peaking factor of 1.28. The non-uniform fuel distribution strategy reduced the power peaking by 75% to a maximum peaking factor of 1.07, which translates to a 28.7% enhancement in operating power (from 738kWth to 950kWth).

The methodological framework presented in this work can be adapted to any single-batch micronuclear reactor core to assess the effects of non-uniform enrichment distribution. As global interest in decentralized and flexible nuclear energy grows, such strategies become increasingly vital in accelerating innovation while maintaining safety margins. The methodology adopted here can also serve as a framework for evaluating other design innovations, particularly in settings where experimental validation is limited.

## Data Availability

Data availability: All data have been described in the text.
