# Correction: Sensitivity-informed framework for enrichment distribution in MNR for thermal performance enhancement

**DOI:** 10.1038/s41598-026-52232-0

**Published:** 2026-05-28

**Authors:** Umair Aziz, Hamda Khan, Zahid Hussain, Khalil Ullah, Imran Shah, Dong-Won Jung

**Affiliations:** 1 Department of Mechatronics Engineering, Air University, Islamabad, Pakistan; 2 Department of Sciences and Humanities, National University of Computer and Emerging Sciences, Islamabad, Pakistan; 3 Faculty of Applied Energy System, Major of Mechanical Engineering, Jeju National University, 102 Jejudaehak-ro, Jeju-Si, 63243 Republic of Korea

Correction to: *Scientific Reports* 10.1038/s41598-026-43564-y, published online 11 March 2026

In the original version of this Article, Imran Shah was omitted as a corresponding author. Correspondence and requests for materials should also be addressed to imranshahswabi@gmail.com.

The original Article has been corrected.

